# Dose-Duration Reciprocity for G protein activation: Modulation of kinase to substrate ratio alters cell signaling

**DOI:** 10.1371/journal.pone.0190000

**Published:** 2017-12-29

**Authors:** Kang-Ling Liao, Charles E. Melvin, Rosangela Sozzani, Roger D. Jones, Timothy C. Elston, Alan M. Jones

**Affiliations:** 1 Departments of Biology, University of North Carolina at Chapel Hill, Chapel Hill, NC, United States of America; 2 Department of Plant and Microbial Biology, North Carolina State University, Raleigh, NC, United States of America; 3 Center for Complex Systems and Enterprises, Stevens Institute of Technology, Hoboken, NJ, United States of America; 4 Department of Pharmacology, University of North Carolina at Chapel Hill, Chapel Hill, NC, United States of America; Indian Institute of Science Education and Research, INDIA

## Abstract

In animal cells, activation of heterotrimeric G protein signaling generally occurs when the system’s cognate signal exceeds a threshold, whereas in plant cells, both the amount and the exposure time of at least one signal, D-glucose, are used toward activation. This unusual signaling property called Dose-Duration Reciprocity, first elucidated in the genetic model *Arabidopsis thaliana*, is achieved by a complex that is comprised of a 7-transmembrane REGULATOR OF G SIGNALING (RGS) protein (AtRGS1), a Gα subunit that binds and hydrolyzes nucleotide, a Gβγ dimer, and three WITH NO LYSINE (WNK) kinases. D-glucose is one of several signals such as salt and pathogen-derived molecular patterns that operates through this protein complex to activate G protein signaling by WNK kinase transphosphorylation of AtRGS1. Because WNK kinases compete for the same substrate, AtRGS1, we hypothesize that activation is sensitive to the AtRGS1 amount and that modulation of the AtRGS1 pool affects the response to the stimulant. Mathematical simulation revealed that the ratio of AtRGS1 to the kinase affects system sensitivity to D-glucose, and therefore illustrates how modulation of the cellular AtRGS1 level is a means to change signal-induced activation. AtRGS1 levels change under tested conditions that mimic physiological conditions therefore, we propose a previously-unknown mechanism by which plants react to changes in their environment.

## Introduction

Extracellular signals such as hormones are perceived by cell-membrane receptors. The existence of an extracellular signal molecule, such as a hormone or a neurotransmitter, causes the receptor to act on molecules internal to the membrane such as the heterotrimeric G protein complex. When activated by the receptor, the heterotrimeric G protein complex, which associates with the receptor on the cytoplasmic side of the membrane, exchanges bound GDP for GTP [1, 2]. The complex is then available to interact with cellular components that confer a particular cell behavior such as muscle contraction or altered metabolism [3–5]. The G protein has an intrinsic GTP hydrolysis activity that returns the G protein to its GDP-bound, inactive state ready to cycle again if the signal remains above its threshold. In animal cells, this G protein cycle is rate limited by guanine nucleotide exchange, a property controlled by the signal and its receptor. However, in plant cells and protists, nucleotide exchange is spontaneous; rather GTP hydrolysis is the rate-limiting step in the G protein cycle. Modulation of the G protein cycle is through a 7-transmembrane receptor that keeps the G protein complex in its inactive state [6–9]. Extracellular signals de-repress activation leading to spontaneous nucleotide exchange and thus activation. The prototype receptor is Arabidopsis Regulator of G Signaling (AtRGS1) and shown to be a shadow detector controlling the efficiency of photosynthesis probably through detection of fixed sugar amount and duration [10]. AtRGS1 is an important G protein element in extracellular glucose signaling and is a strong candidate as the extracellular glucose receptor of co-receptor [4, 11–16].

One mechanism by which plant cells de-repress the G protein cycle is to internalize AtRGS1 from the cell surface, leaving behind the G protein complex to self- activate [4]. Activation is quantitated using cells expressing AtRGS1 tagged with fluorescent YFP. This data was used to fit a mathematical model describing an emergent property whereby a low dose of signal presented to the cell over a long period of time activated as well as a high concentration presented as a short pulse. This unique property was designated Dose-Duration Reciprocity [15]. The mathematical model predicted that the plant G protein system included two different kinases operating with different time constants and sensitivity to glucose. These two kinases were identified as WITH NO LYSINE (WNK) kinases, WNK1 and WNK8/10 where WNK1 (kinase 2) controls the slow reaction and WNK8 and WNK10 kinases (kinase 1) were assumed to be redundant in controlling the fast reaction however they may not be. These WNK kinases phosphorylate AtRGS1, a key reaction controlling the amount of the receptor, therefore it is essential to incorporate our understanding of the total amount of AtRGS1 and the ratio of kinases to this receptor to predict signal-induced behavior involving the G protein elements.

The effect of receptor levels on signal-induced behavior is not new. In 1957, Nickerson applied histamine to a guinea pig ileum preparation to show that a maximum contraction was produced when agonist occupied only a small percent of histamine receptors [6]. Similar behavior occurred in the rabbit aortic strips [17]. Consequently, by the 1970’s, the concept of “spare receptor” was evoked to explain why the half-maximal effective concentration (EC_50_) of a hormone was lower than the concentration causing half maximal receptor occupancy (Kd) [18]. In these cases, the rate-limiting step occurs downstream to receptor binding. However, while a rate-limiting step distil to receptor binding was easy enough to understand at the time, a different emergent systems property for spare receptors was not understood. Specifically, early researchers noted that as the amount of receptor increased, the system became more sensitive.

To understand how this occurs, consider the following hormone interaction:

$$H+R\overset{{}_{{}_{k_{1}}}}{\underset{k_{2}}{⇄}}HR$$

where [H], [R], and [HR] are concentrations of hormone, unbound receptor, and bound receptor, respectively, and the association and dissociation rates *k*_1_ and *k*_2_. At the equilibrium, we have the relation that [HR] = (*k*_1_/*k*_2_) [H][R], which means that the more spare receptor R present, the more HR will form at a relatively lower [H]. Consequently, “spare receptors” are not actually spare. Controlling the amount of receptor is a mechanism to control sensitivity.

A similar behavior may occur in G protein activation via regulation of the AtRGS1 pool size, therefore we investigated whether the amount of the total AtRGS1 pool size changes the responsiveness to glucose in Arabidopsis. To quantitate this, we used AtRGS1-YFP endocytosis because it is the current standard method used to report G protein activation. Unfortunately, expression of AtRGS1-YFP, by its nature, increases the pool of AtRGS1 and thus is a perturbation on the system [15]. Therefore, in order to understand how AtRGS1 modulates G activation in the unperturbed state, we determined how in quantitative terms that the total level of AtRGS1 affects the system responsiveness to glucose. We showed that even a modest change in the AtRGS1 pool size changes responsiveness from linear to non-responsive or vice versa depending on the direction of change in pool size. We found physiological conditions where the AtRGS1 pool size changes suggesting a mechanism for natural control of glucose signaling.

## Materials and methods

### Plant growth and genotypes

Stable lines expressing YFP driven by the 35S promoter, namely, 35S::AtRGS1-YFP (denoted as 35S-YFP) and AtRGS1 tagged with YFP driven by the native RGS1 promoter (denoted as RGS1-YFP) were used in the AtRGS1 endocytosis and AtRGS1 expression level experiments.

Arabidopsis seeds expressing AtRGS1-YFP from its native promoter or YFP from the 35S viral promoter were sterilized with ethanol (first with 70%, 10 min, 95% 10 min). Ten to 20 seeds were then sown on 1-mL liquid 1/4 X Murashige and Skoog (MS) medium without sucrose in 24-well plates and stratified at 5°C for 2 days, followed by 2 hours light, then grown in darkness at 27°C for 4 days. For best results, keep the plates kept in darkness but move them to the microscope room on the third day to acclimate. Homozygous *wnk1*, *wnk8-2*, and *wnk10-1* null mutants were generated from T-DNA insertion mutants (SALK_015778, SALK_024887, and SALK_012899) [3]. All DNA-insertion lines were made homozygous and the T-DNA insertions were confirmed by PCR of genomic DNA with the following table for primer sets (Table 1).

**Table 1:** Primers used for genotyping.

Gene	Primer
*wnk1*_RP	CGCAAGACATTCTTCGAATTC
*wnk1*_LP	GGGAATCAAGGAGAGGTCAAG
*wnk8*-2_RP	TACTCCTGAATTCATGGCACC
*wnk8*-2_LP	CAGCAGATCTTGGAAGGACTG
*wnk10*_RP	TGCTCTTCTGCTAAAAGCAGC
*wnk10*_LP	GGGTCCATTCCTCTCTCTCAG

### Experimental design and image acquisition

Before taking images, the seedling was moved to a well with 2-mL sterile water for 5–10 minutes to wash out the MS salts. For the glucose-induced endocytosis experiment, after this wash the seedlings (wild type, *wnk*8/10 null mutant, and *wnk*1 null mutant) were gently placed into another well containing D-glucose, with a dwell time of 1 min between each seedling, and then moved onto one slide for image acquisition. For the AtRGS1 endocytosis fraction, the image was taken at the vertical optical sections (i.e. Z stacks acquired) of hypocotyl epidermal cells located approximately 3–4 mm from the cotyledon. For AtRGS1-YFP fluorescence intensity quantitation, seedlings were washed and moved to a slide with 100 mM NaCl, 100 mM sorbitol, 100 mM flg22, water, or 2% D-glucose, and left on the microscope stage over the time course of image acquisition. The hypocotyl epidermal cells imaged were taken at the vertical optical sections (i.e. Z stacks acquired) located 2–4 mm below the cotyledons.

### Microscopy

A Zeiss LSM710 confocal laser scanning microscope with a C-Apochromat 40X 1.2N.A. water immersion objective was used to quantitate the proportion of internalized AtRGS1. The YFP fluorescence was excited by a 514-nm argon laser and the photomultiplier detector was set between 526 nm and 569 nm for quantification. The proportion of internalized AtRGS1 was analyzed by Image J. Illumination was as short as possible to avoid heating or photobleaching.

To quantitate the relative amount of AtRGS1 over time after treatment, imaging was performed using light sheet fluorescence microscopy (Zeiss Lightsheet Z.1) and the Multi-sample Arabidopsis Growth and Imaging Chamber (MAGIC) (de Luis Balaguer et al. 2016). Lines 35S-YFP and RGS1-YFP were prepared as described in de Luis Balaguer et al. [19]. 35S-YFP roots were used as the control for the D-glucose treatment. Six and eight roots of the 35S-YFP and RGS1-YFP, respectively, were loaded into a MAGIC device and imaged every 15 minutes in deionized water (first 15’) and then with a 6% D-glucose solution (from 15’ to 165’) (Sigma G8270). Due to differing optical properties of the deionized water and the D-glucose solution, the lightsheet was realigned between treatments. Laser (488 nm) and bright field intensities remained constant between the deionized water and D-glucose phases of the experiment. The Lightsheet Z.1 native incubator was used to keep the imaging chamber at 22 ºC throughout the experiment. For the analysis, the maximum intensity projections were generated using Zeiss Zen Black edition (Zen 2014 SP1) and exported as tiff images using Zen 2.3 lite. The average pixel intensity of the images was processed in MATLAB (See Supplement-Matlab Code, S1 File). Briefly, a mask was applied to remove the image background of the maximum intensity projections of each root, leaving only pixels containing root. The average intensity of the pixels corresponding to the root were calculated.

### Mathematical model

#### Variables of the model (Table 2)

**Table 2:** Variables of the model.

Variable	Description
*x*_1_	free AtRGS1
*x*_2_	AtRGS1:G*α*^*GDP*^*βγ*
*x*_3_	AtRGS1:G*α*^*GTP*^*βγ*
*x*_4_	AtRGS1:G*α*^*GDP*^
*x*_5_	AtRGS1:G*α*^*GTP*^
*x*_6_	G*α*^*GDP*^*βγ*
*x*_7_	G*α*^*GTP*^*βγ*
*x*_8_	G*α*^*GDP*^
*x*_9_	G*α*^*GTP*^
*x*_10_	G*βγ*
*x*_11_	phosphorylated AtRGS1
*x*_12_	internalized AtRGS1
*x*_13_	D-glucose receptor
*x*_14_	Kinase 2 (WNK1)
*x*_15_	Kinase 1 (WNK8/10)
L	D-glucose

#### Glucose-input model

$$\frac{dx_{1}}{dt}=-k_{17}x_{1}x_{7}-k_{18}x_{1}x_{9}-k_{12}x_{1}+k_{24}x_{5}+k_{25}x_{3}+k_{27}x_{12}$$ (1)

$$\frac{dx_{2}}{dt}=k_{4}x_{3}+k_{8}x_{4}x_{10}-k_{6}x_{2}-k_{16}x_{2}$$ (2)

$$\frac{dx_{3}}{dt}=k_{6}x_{2}-k_{4}x_{3}-k_{11}x_{3}\frac{x_{13}^{k_{14}}}{k_{26}^{k_{14}}+x_{13}^{k_{14}}}-k_{25}x_{3}+k_{17}x_{1}x_{7}+k_{13}x_{5}x_{10}$$ (3)

$$\frac{dx_{4}}{dt}=k_{4}x_{5}-k_{6}x_{4}-k_{8}x_{4}x_{10}+k_{16}x_{2}$$ (4)

$$\begin{array}{l} \frac{dx_{5}}{dt}=k_{6}x_{4}+k_{11}x_{3}\frac{x_{13}^{k_{14}}}{k_{26}^{k_{14}}+x_{13}^{k_{14}}}-k_{4}x_{5}-k_{3}x_{5}(x_{14}+x_{15})+k_{30}x_{11}+k_{18}x_{1}x_{9}-k_{2}x_{5}\\ \;-k_{24}x_{5}-k_{13}x_{5}x_{10} \end{array}$$ (5)

$$\frac{dx_{6}}{dt}=k_{5}x_{7}+k_{7}x_{8}x_{10}-k_{6}x_{6}-k_{28}x_{6}$$ (6)

$$\frac{dx_{7}}{dt}=k_{6}x_{6}-k_{5}x_{7}-k_{10}x_{7}-k_{17}x_{1}x_{7}+k_{9}x_{9}x_{10}+k_{25}x_{3}$$ (7)

$$\frac{dx_{8}}{dt}=k_{5}x_{9}+k_{28}x_{6}-k_{6}x_{8}-k_{7}x_{8}x_{10}$$ (8)

$$\frac{dx_{9}}{dt}=k_{20}x_{11}+k_{6}x_{8}+k_{10}x_{7}-k_{5}x_{9}-k_{18}x_{1}x_{9}-k_{9}x_{9}x_{10}+k_{2}x_{5}+k_{24}x_{5}$$ (9)

$$\frac{dx_{10}}{dt}=k_{10}x_{7}+k_{11}x_{3}\frac{x_{13}^{k_{14}}}{k_{26}^{k_{14}}+x_{13}^{k_{14}}}-k_{8}x_{4}x_{10}-k_{7}x_{8}x_{10}-k_{9}x_{9}x_{10}+k_{28}x_{6}-k_{13}x_{5}x_{10}+k_{16}x_{2}$$ (10)

$$\frac{dx_{11}}{dt}=k_{3}x_{5}(x_{14}+x_{15})-k_{20}x_{11}-k_{30}x_{11}$$ (11)

$$\frac{dx_{12}}{dt}=k_{2}x_{5}+k_{12}x_{1}+k_{20}x_{11}-k_{27}x_{12}$$ (12)

$$\frac{dx_{13}}{dt}=k_{15}(L-x_{13})$$ (13)

$$\frac{dx_{14}}{dt}=\left\{ \begin{array}{l} k_{1}(\frac{k_{21}x_{10}^{2}}{k_{22}^{2}+x_{10}^{2}}-x_{14}),\;\mathrm{two-kinase}\;\mathrm{model}\\ 0,\;\mathrm{one-kinase}\;\mathrm{model} \end{array}\right.$$ (14)

$$\frac{dx_{15}}{dt}=k_{19}(\frac{k_{23}x_{10}^{2}}{k_{29}^{2}+x_{10}^{2}}-x_{15})$$ (15)

#### Conservation laws

For the model in Eqs (1–15), summing all equations related to the AtRGS1 complex, one obtains:

$$(\frac{dx_{2}}{dt}+\frac{dx_{3}}{dt}+\frac{dx_{4}}{dt}+\frac{dx_{5}}{dt})+(\frac{dx_{1}}{dt}+\frac{dx_{11}}{dt}+\frac{dx_{12}}{dt})=0,\;\mathrm{for}\;\mathrm{all}\;t\geq 0,$$ (16)

which represents that the total level of AtRGS1 over time. Similarly, summing up all equations related to GPA1 and AGB and AGG complexes, one obtains:

$$(\frac{dx_{2}}{dt}+\frac{dx_{3}}{dt}+\frac{dx_{4}}{dt}+\frac{dx_{5}}{dt})+(\frac{dx_{6}}{dt}+\frac{dx_{7}}{dt}+\frac{dx_{8}}{dt}+\frac{dx_{9}}{dt})+\frac{dx_{11}}{dt}=0,\;\mathrm{for}\;\mathrm{all}\;t\geq 0,$$ (17)

and

$$\frac{dx_{2}}{dt}+\frac{dx_{3}}{dt}+\frac{dx_{6}}{dt}+\frac{dx_{7}}{dt}+\frac{dx_{10}}{dt}=0,\;\mathrm{for}\;\mathrm{all}\;t\geq 0,$$ (18)

which represents the total amount of GPA1 and AGB and AGG complexes, respectively, over time.

## Results

### The importance of the AtRGS1 substrate to WNK kinase ratio

**Fig 1:**
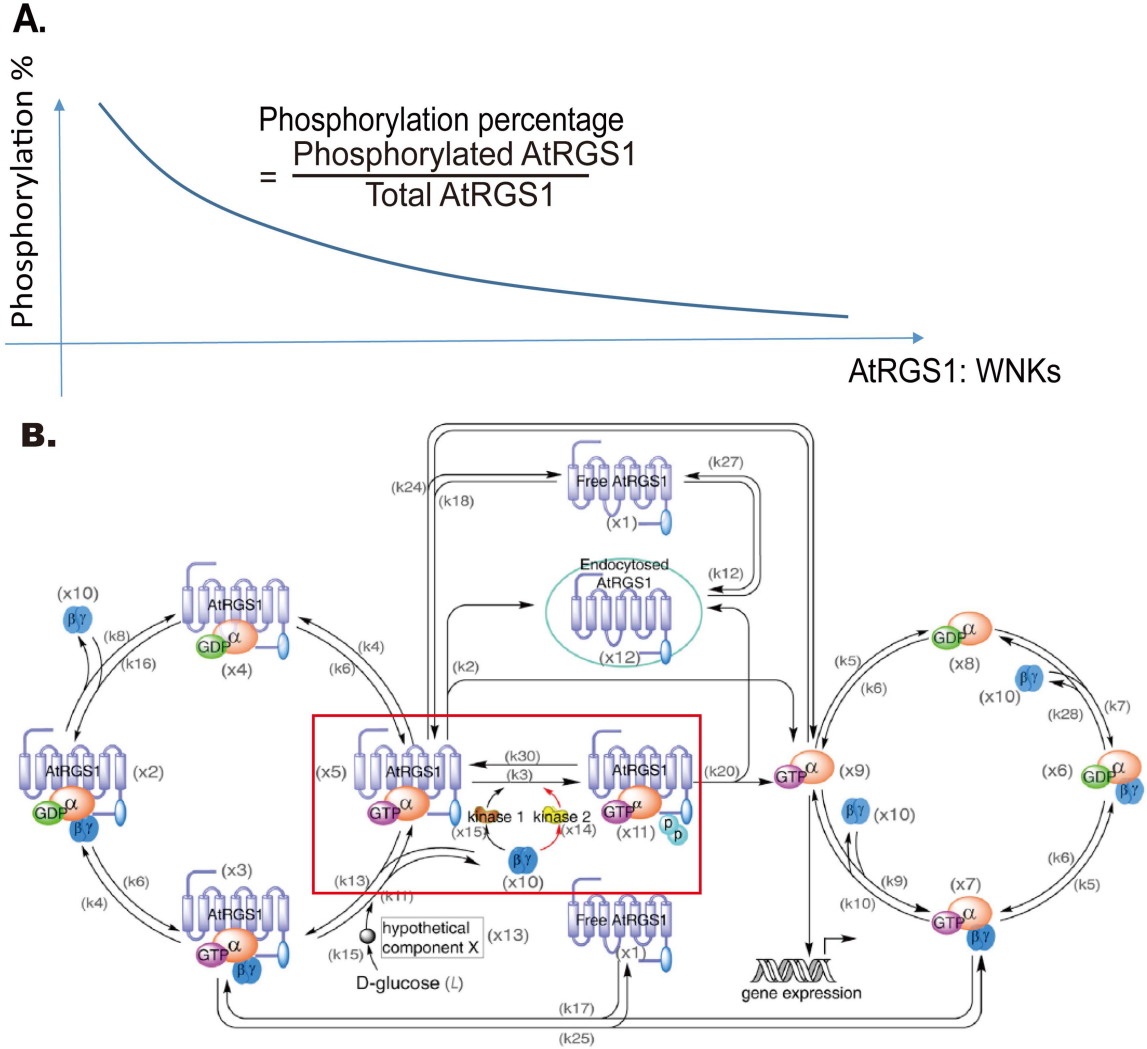
Hypothesis: AtRGS1 phosphorylation proportion and endocytosis depend on the AtRGS1 level. The hypothesis is that the AtRGS1 level affects the phosphorylation percentage in G protein signaling pathway and the level of activation of the G protein pathway. **(A).** The relationship between phosphorylated AtRGS1 and the ratio AtRGS1:WNK kinases. The natural variation in expression levels among transformed cells will be used to test this hypothesis. **(B).** Network of G protein signaling pathway. This model is taken from Fu, et al 2014. The added red box marks the process affected by our hypothesis.

Our hypothesis is that the total AtRGS1 level affects glucose responsiveness as determined by AtRGS1-YFP endocytosis because the competition between WNKs kinases depends on the AtRGS1 pool size. The G protein signaling pathway requires kinases having different reaction rates in order to respond to signal intensity and duration [15]. As for any enzyme-substrate relationship, the ratio of AtRGS1 and WNKs kinases is important. Fig 1A illustrates our hypothesis that the phosphorylation proportion, a proxy for activation, is a function of the ratio of AtRGS1-YFP level and WNKs kinases. A high AtRGS1 level predicts a low phosphorylation proportion, thus activation, whereas a low AtRGS1 pool size with fixed amount of the WNKs kinases predicts a high proportion of activation. Therefore, we chose to simplify by modeling only the relevant kinase-AtRGS1 reactions in the Dose-Duration Model as shown in the red box in Fig 1B. Variables used in the model are provided in Table 2.

**Fig 2:**
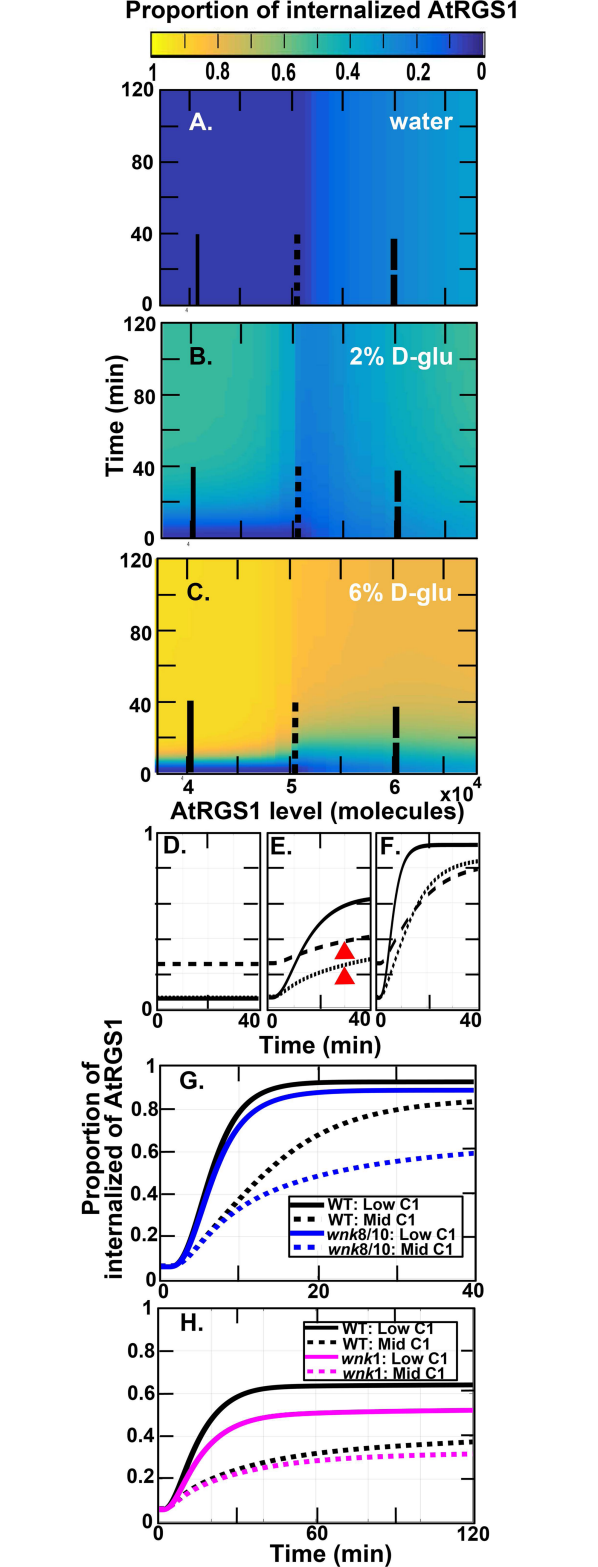
Relationship between AtRGS1 endocytosis and the AtRGS1 level in wild type. **(A-C).** The heat map of the proportion of internalized AtRGS1 under water, 2%, and 6% D-glucose treatment for the cells with AtRGS1 level where *C*1∈[3.7×10^4^, 6.8×10^4^] molecules, by numerical simulation of the mathematical model and setting used by [15]. The color bar shown on the top represents the proportion of internalized AtRGS1 value ranging from blue (0.00) to yellow (1.00). In **(A-C)**, the proportion of internalized AtRGS1 increases as time increases, however, the relation between the proportion of internalized AtRGS1 and AtRGS1 level (*C*1) is nonlinear. **(D-F).** AtRGS1 endocytosis dynamics within the first 40 minutes of water- and glucose-treated cells with low, moderate, and high AtRGS1 levels in panels (A-C). **D.** Water treated controls. **E.** 2% D-glucose. **F.** 6% D-glucose. Curves are for different AtRGS1 levels: Solid line (*C*1 = 4×10^4^ molecules), dotted line (*C*1 = 5×10^4^ molecules), and dashed lines (*C*1 = 6×10^4^ molecules) as illustrated in panels A-C. Red arrow heads mark the tested time points discussed in Fig 3. **(G-H).** Comparison of the proportion of internalized AtRGS1 in wild type, *wnk*8/10, and *wnk*1 null mutants under different AtRGS1 levels after high and low D-glucose concentration treatment. **(G).** Wildtype, black solid (*C*1 = 4×10^4^ molecules) and dotted (C1 = 5×10^4^ molecules); *wnk8/10* mutant, blue solid (*C*1 = 4×10^4^) and dotted (*C*1 = 5×10^4^) curves represent the proportion of internalized AtRGS1 after 6% D-glucose treatment over 40 minutes. (**H**)**.** Wildtype, black solid (*C*1 = 4×10^4^ molecules) and dotted (*C*1 = 5×10^4^ molecules); *wnk1* mutant, magenta solid (*C*1 = 4×10^4^) and dotted (*C*1 = 5×10^4^) curves represent the proportion of internalized AtRGS1 after 2% D-glucose treatment over 120 minutes.

Next, we explored the relationship of AtRGS1 levels on glucose-induced activation of G signaling in the model [15]. This model (Fig 1B) is a closed system obeying conservation laws for AtRGS1, AtGPA1, and AGB/AGG complexes. The constant amount of AtRGS1, AtGPA1, and AGB1/AGG are denoted as *C*1, *C*2, and *C*3, respectively. To test whether AtRGS1 pool size (i.e., *C*1) affects D-glucose-induced AtRGS1-YFP endocytosis, we simulated the proportion of internalized AtRGS1 at different AtRGS1 levels, i.e., at different *C*1 values. As shown in Fig 2, the proportion of internalized AtRGS1 was sensitive to *C*1. Fu and co-workers [5] set C1 = 5.2 × 10^4^ molecules in their simulations. Therefore, we randomly chose initial values such that

$$C1\in [3.7\times 10^{4},\;6.8\times 10^{4}]\;\mathrm{molecules}$$

and *C*1 = *C*2 (as the set in [5]) and *C*3 = 5.1 × 10^4^ molecules for wild type (as the set in [5]), *wnk*1 null mutant, and *wnk*8/10 null mutant.

The proportion of AtRGS1 internalization and thus the amount of G signaling activation after glucose depends on *C*1. The raw data supporting this is illustrated by heat maps in Fig 2A and 2C displaying the proportion of internalized AtRGS1 of wild type at 0% (water only), 2%, and 6% D-glucose when *C*1∈[3.7×10^4^, 6.8×10^4^] molecules. Another way to illustrate this property is to plot the proportion of AtRGS1 over time at just three members of the *C*1 set. The solid, dotted, and dashed lines in Fig 2A and 2C denote, respectively, a low *C*1 value (i.e., solid line *C*1 = 4×10^4^ molecules), a medium *C*1 value (i.e., dotted line *C*1 = 5×10^4^ molecules), and a high *C*1 value (i.e., dashed line *C*1 = 6×10^4^ molecules). Fig 2D and 2F plot the values represented by the respective heat map in Fig 2A and 2C as a function of time after water (no-glucose control) or the addition of glucose. Without glucose, the values remained constant, but the constant depended on *C*1 (cf. Fig 2A and 2D). Upon glucose stimulation, the relation between *C*1 and the proportion of internalized AtRGS1 became nonlinear in that the proportion of internalized AtRGS1 did not always increase as *C*1 increased. For example, after 2% glucose treatment (Fig 2E), cells with lower AtRGS1 level had a lower proportion of internalized AtRGS1 than cells with higher AtRGS1 level before 10 minutes, but this relation reversed after 10 minutes (the solid and dashed curves in Fig 2B and 2E). A similar behavior occurred after 6% glucose treatment (cf. Fig 2C and 2F). This indicates that the system is sensitive to *C*1; a difference in *C*1 as small as 20% altered the glucose responsive. Moreover, as shown in Fig 2D and 2F, at the higher AtRGS1 level (i.e., dashed curves) the system was less sensitive to glucose treatment because the system was close to saturation, such that glucose induced a lower proportion of internalized AtRGS1 than the system having a lower AtRGS1 level (i.e., solid curves).

Previous published works have not taken into consideration the effect of *C*1 or the ratio of AtRGS1:WNK on glucose activation. For example, to fit the original model, Fu and coworkers [15] used experimental data obtained from a genetically stable plant line that constitutively overexpressed AtRGS1-YFP but for testing and subsequent validation, they used data obtained from transient expression in the *wnk* null mutants. This difference is important because transient expression generally produces a higher AtRGS1 level than expression in genetically stable lines. Fu et al reported that the *wnk*1 null mutation only reduced endocytosis at low dose/high duration of glucose compared to wild type, whereas the *wnk*8/10 null mutations only reduced endocytosis at high dose/short duration of glucose.

As shown in Fig 1A, it is now clear that endocytosis is dependent on the AtRGS1:WNK kinase ratios. Therefore, whether or not the *wnk* null mutations alter the sensitivity to glucose also depends on the AtRGS1 level and which WNK kinases is genetically ablated. This was tested. For the *wnk8/10* null mutations, the difference in the proportion of internalized AtRGS1 between wild type and the *wnk*8/10 mutant under high glucose concentration (i.e., 6% glucose) was small when the AtRGS1 level was low (Fig 2G, black and blue solid curves, 6%), but this difference increased as the AtRGS1 level increased (Fig 2G, black and blue dotted curves). However, this relation between wild type and *wnk*8/10 mutant was reversed under low glucose concentration (i.e., 2% glucose) in S1A Fig. For the *wnk1* null mutation, the relation between wild type and the *wnk1* null mutant reversed under low glucose concentration, i.e., 2% glucose (Fig 2H). A similar relationship between wild type and the *wnk*1 null mutant occurred at high glucose concentration, i.e., 6% glucose, in S1B Fig. Note that the difference in the proportion of internalized AtRGS1 between wild type and *wnk*1 null mutant decreased as the AtRGS1 level increased (Fig 2H, black and magenta dotted curves). The AtRGS1 endocytosis dynamics in the *wnk*8/10 and *wnk*1 null mutants with *C*1∈[3.7×10^4^, 6.8×10^4^] molecules under different D-glucose treatment are provided in S2 Fig. The results of this simulation supports our hypothesis that the AtRGS1 level affects the cellular response to glucose and also emphasizes the importance of knowing the expression level of the AtRGS1-YFP reporter in making conclusions on activation of the G protein pathway.

#### G signaling dynamics under different AtRGS1 and WNK kinase levels

**Fig 3:**
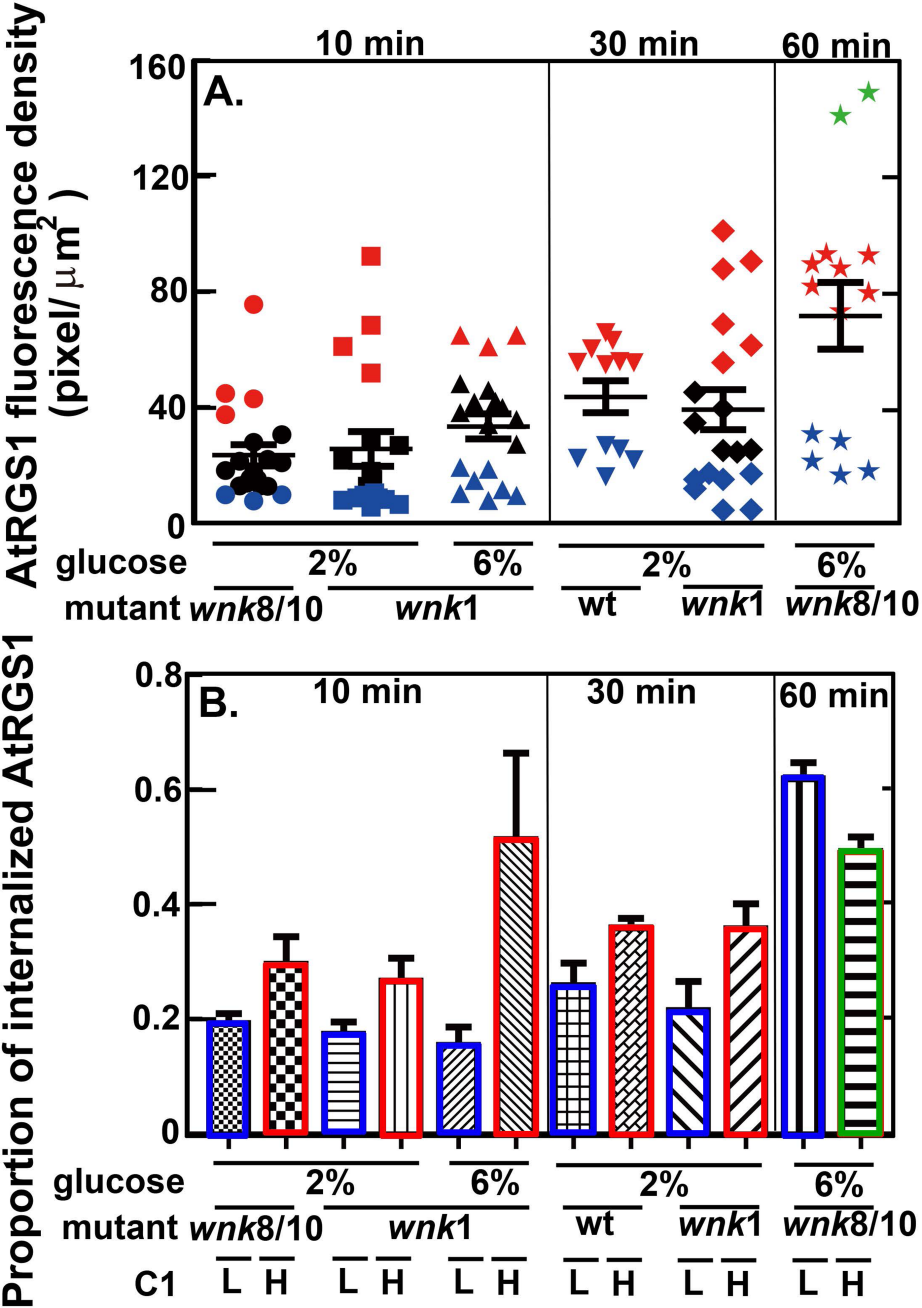
Experimental testing of the mathematical model. ***C*1 affects glucose-induced AtRGS1 endocytosis dynamics.** Proportion of glucose-induced AtRGS1-YFP endocytosis that occurred with different starting levels of AtRGS1 binned based on YFP fluorescence intensity. For these experiments, AtRGS1-YFP was driven by the 35S viral promoter and the natural variation in expression level was utilized. **(A).** The AtRGS1 fluorescence intensity distribution for wild type and *wnk* null mutants under different D-glucose treatments and times were binned accordingly: green, highest AtRGS1 intensity; red, moderate intensity; black intermediate intensity; blue, lowest intensity. Quantitation was performed using cells from the indicated genotypes. Each symbol represents the AtRGS1 fluorescence intensity for a single cell. Intensity is correlated with AtRGS1 level (*C*1 in the conservation law). **(B).** The proportion of internalize AtRGS1at three times corresponded to the cells with binned high and low AtRGS1 fluorescence intensity shown in panel A. Left panel, *wnk*8/10 null mutant under 2% glucose treatment, *wnk*1 null mutant under 2% glucose treatment, and *wnk*1 null mutant under 6% glucose treatment for 10 min; Middle panels, wild type and *wnk*1 null mutant under 2% glucose treatment for 30 min; Right panel, *wnk*8/10 null mutant under 6% glucose treatment for 60 min. Each group is separated into four groups: high, moderate, intermediate, and low AtRGS1 intensity, which were marked by green, red, black, and blue symbols based on the intensity shown in A. Experimental results fit the model predictions and are discussed.

To test the model, we measured the proportion of internalized AtRGS1 in cells with different AtRGS1 levels (Fig 3A). This experiment was performed in the following way: First, the natural variation of AtRGS1-YFP fluorescence densities was separated into four groups: relatively high density (green symbols), medium density (red symbols), intermediate (black symbols), and low density (blue symbols). Second, cells were treated with glucose and the proportion of internalized AtRGS1 was quantified.

The model predictions in Fig 2 (for wild type) and S1 Fig (for *wnk*8/10 and *wnk*1 null mutants) showed that the relationship between endocytosis and the AtRGS1 level was nonlinear, namely, cells expressing the higher AtRGS1 level did not always have a higher proportion of AtRGS1-YFP endocytosis than the cells expressing the lower level. This was validated by the experimental results shown in Fig 3B in which AtRGS1 fluorescence intensity correlated with AtRGS1 level (Fig 3A). Three key observations validate the model simulation: **1)** In the *wnk*8/10 mutant treated with 6% glucose for 60 min, there was a lower proportion of internalized AtRGS1 in cells with higher AtRGS1 levels compared to lower levels as predicted (S1F Fig; cf. solid to dash curves). **2)** Regardless of the genotype at a low dose of glucose, there was a higher proportion of internalized AtRGS1 in cells with a higher level of AtRGS1 (Fig 3, 2% values) as predicted (Fig 2E, red arrow heads [cf. dash vs dotted curves]; S1K Fig, red arrowheads [dashed vs dotted curves]; S1E Fig, red arrowheads [dashed vs. dotted curves]). **3)** In the *wnk1* mutant treated with 6% glucose for 10 min, there was a higher proportion of internalized AtRGS1 in cells with a higher level of AtRGS1 (S1L Fig, red arrowheads [dashed vs dotted curves]).

This nonlinear relation between AtRGS1 level and glucose activation of G signaling may be caused by saturation of the AtRGS1-kinase reaction. For cells with a lower AtRGS1 level (i.e., the solid curves in Fig 2 and S1 Fig), the WNK kinases may have a better efficiency or sensitivity to interact with AtRGS1 and hence the cells internalize more AtRGS1 than the cells with a higher AtRGS1 level.

### AtRGS1 levels change under different physiological conditions

**Fig 4:**
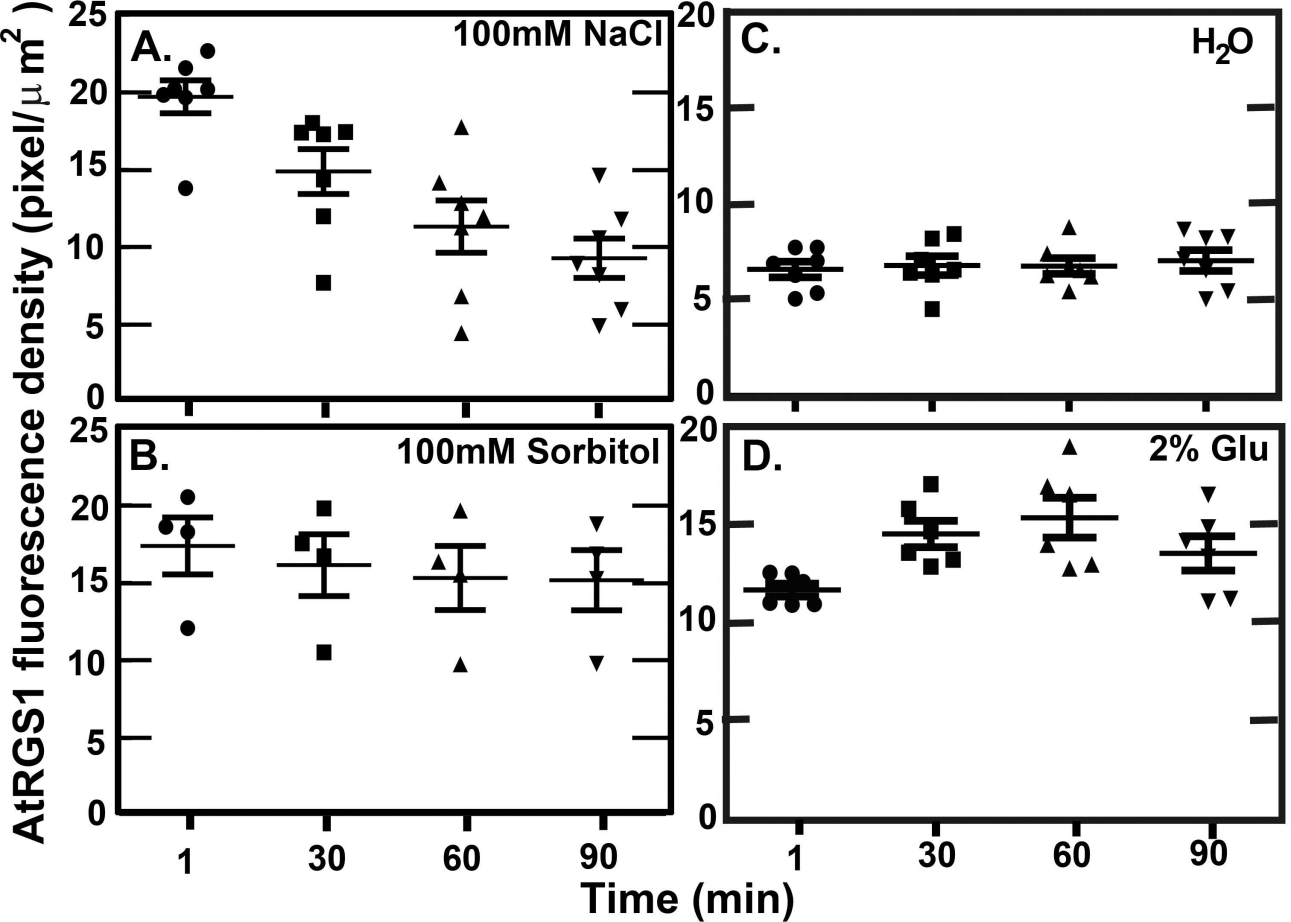
Two physiological conditions alter the level of AtRGS1 (*C*1 in the model). Panels show the change in AtRGS1 intensity over 90 minutes after treatment with 100 mM NaCl treatment **(A)**, 100 mM D-Sorbitol **(B)**, water **(C),** and 110 mM (2%) D-glucose **(D)**. The image was taken every 30 minutes at the same location of the same seedlings over 90 min in (A-D). In (A), after 100 mM NaCl treatment, the AtRGS1-YFP intensity significantly decreased over 90 minutes. In (B) and (C), the AtRGS1-YFP intensity in the controls (i.e., 100 mM D-Sorbitol and water) was constant over 90 minutes. In (D), after 110 mM D-glucose treatment, the AtRGS1 density significantly increases after 60 minutes treatment. This suggests that the nutritional state of the plant cell affects the AtRGS1 level. For these experiments, AtRGS1-YFP was driven by the 35S viral promoter.

While the previous results showed that glucose sensitivity and the behavior of the cell to activate G signaling is sensitive to the AtRGS1 level, it is not known if AtRGS1 levels actually vary in nature and if so, by how much. Therefore, to determine whether the AtRGS1 level is regulated by external stimuli under physiological conditions, we subjected decapitated (cotyledons) seedlings expressing AtRGS1-YFP under the control of the viral 35S promoter to 100 mM NaCl (Fig 4A), 100 mM sorbitol (Fig 4B), water (Fig 4C), 2% D-glucose (Fig 4D), and 100 mM flg22 (S3 Fig) to measure AtRGS1 fluorescence intensity change. Cotyledons were removed to lower the sugar baseline. NaCl as an external stimulus was chosen because it is a major threat to modern agriculture [20] and because it is known that glucose attenuates the deleterious effect of NaCl on seedlings [21]. Slightly saline soils have a salt concentration of 50–150 mM and the sugar concentration of plant cells ranges from femtomolar to high millimolar [22]. Sorbitol was included to measure the effect of osmosis. The water-only control and sorbitol treatment showed that the level of AtRGS1-YFP was constant over the test period. NaCl caused a 2-fold reduction in the AtRGS1-YFP level by 90 minutes and glucose caused a 1.5-fold increase by 60 minutes. The NaCl-induced effect on AtRGS1 level is consistent with the observation that glucose rescues plant growth under salt stress [21], because the reduction of the AtRGS1 level by NaCl increased the sensitivity of endocytosis to glucose (namely, the proportion of internalized AtRGS1 is predicted to increase (see Fig 2). This result also suggest that there is a de-sensitization mechanism to glucose whereby glucose increases the level of AtRGS1 over time leading to reduced glucose sensitivity. The bacterial plant pathogen elicitor, flg22, a 22-amino acid peptide from the bacterial flagellin induces RGS1 endocytosis [23, 24]. We tested if a high concentration (100 mM) caused a change in the AtRGS1 level and did not observe a statistically-significant difference (p-value was around 0.02) over the 30 minute time course (S3 Fig).

**Fig 5:**
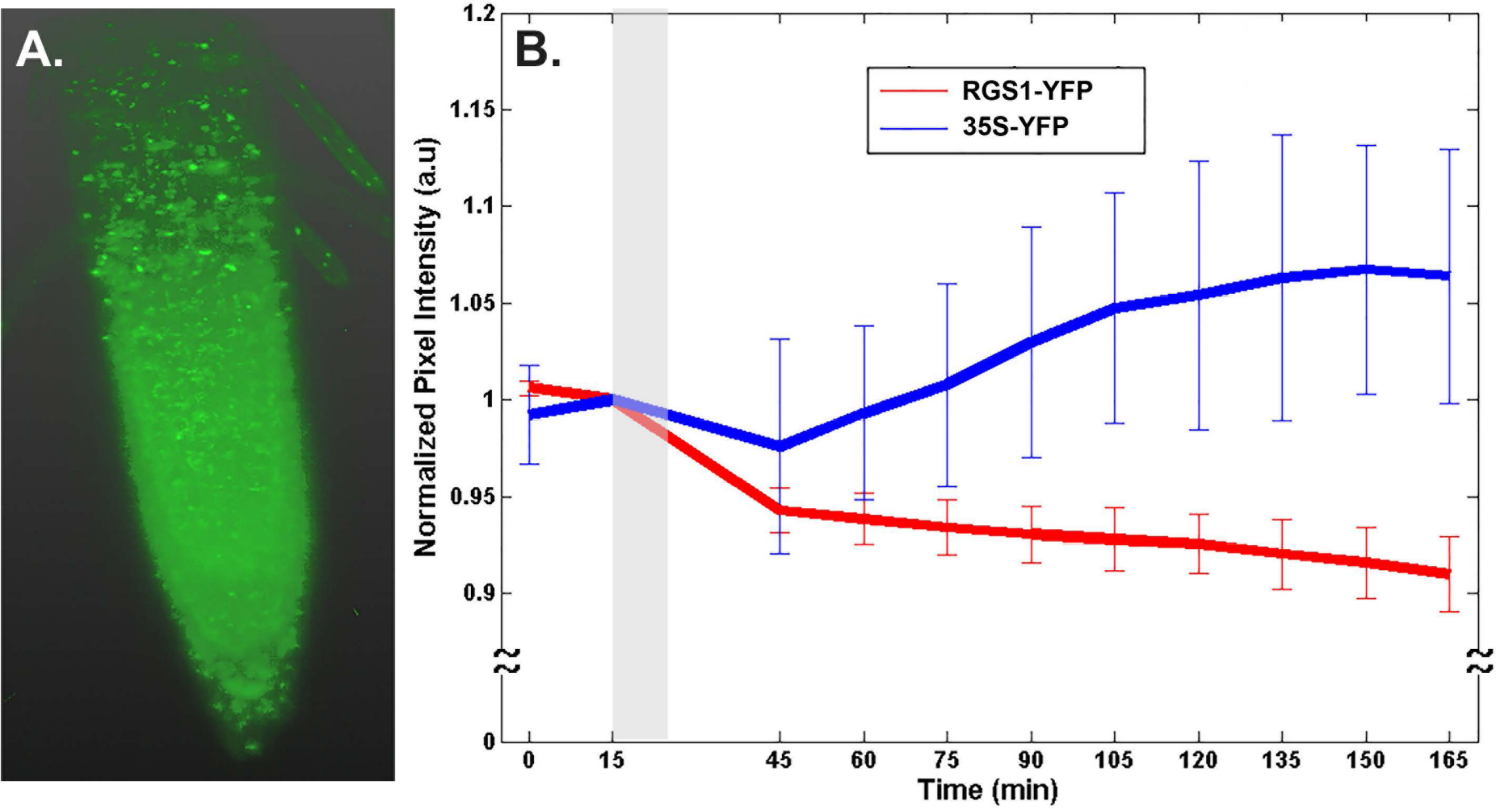
D-glucose treatments decrease the level of AtRGS1. **(A).** 3D reconstruction of AtRGS1-YFP translational fusion within the root tip as imaged in the Zeiss Lightsheet Z.1. **(B).** Quantitation of the normalized pixel intensity of 6 and 8 biological replicates of 35S-YFP and RGS1-YFP, respectively. Grey bar represents the D-glucose treatment time and error bars represent standard deviations.

An alternative method was used to quantitate changes in AtRGS1-YFP level after glucose treatment. Lightsheet microscopy has the advantage in that photobleaching is negligent. Moreover, the increased detection sensitivity of Lightsheet microscopy enabled us to quantitate changes in AtRGS1-YFP when expressed by its native gene promoter (Fig 5) compared to higher expression by the 35S promoter (Fig 4) required to detect AtRGS1-YFP by confocal microscopy (Fig 4). The root tip of a seedling expressing AtRGS1-YFP at its native level is shown in Fig 5A. The intensity of YFP was captured every 15 min. As expected due to ectopic expression by the 35S promoter, the YFP control, although variable, showed an increase over time (P = 0.016). During glucose application (grey zone, Fig 5), there was a decrease in both the 35S-YFP control and the RGS1-YFP lines. This a solution-changing artifact. There was no difference in the slope before vs. after glucose application for the 35S-YFP control (P = 0.5). In contrast to the 35S-YFP control, the intensity of the AtRGS1-YFP protein was much less variable and clearly decreased over the entire time course (P = 1.69632×10^−9^). After switching from water to 6% glucose, the decrease in the intensity of AtRGS1-YFP was 2-3-fold less than during the water treatment (P = 0.1). Thus is contrast to hypocotyl cells expressing a higher proportion of AtRGS1 where glucose causes a decrease in the AtRGS1-YFP pool. Root cells expressing the native level of AtRGS1 stabilize the AtRGS1-YFP pool over 2 hours.

**Fig 6:**
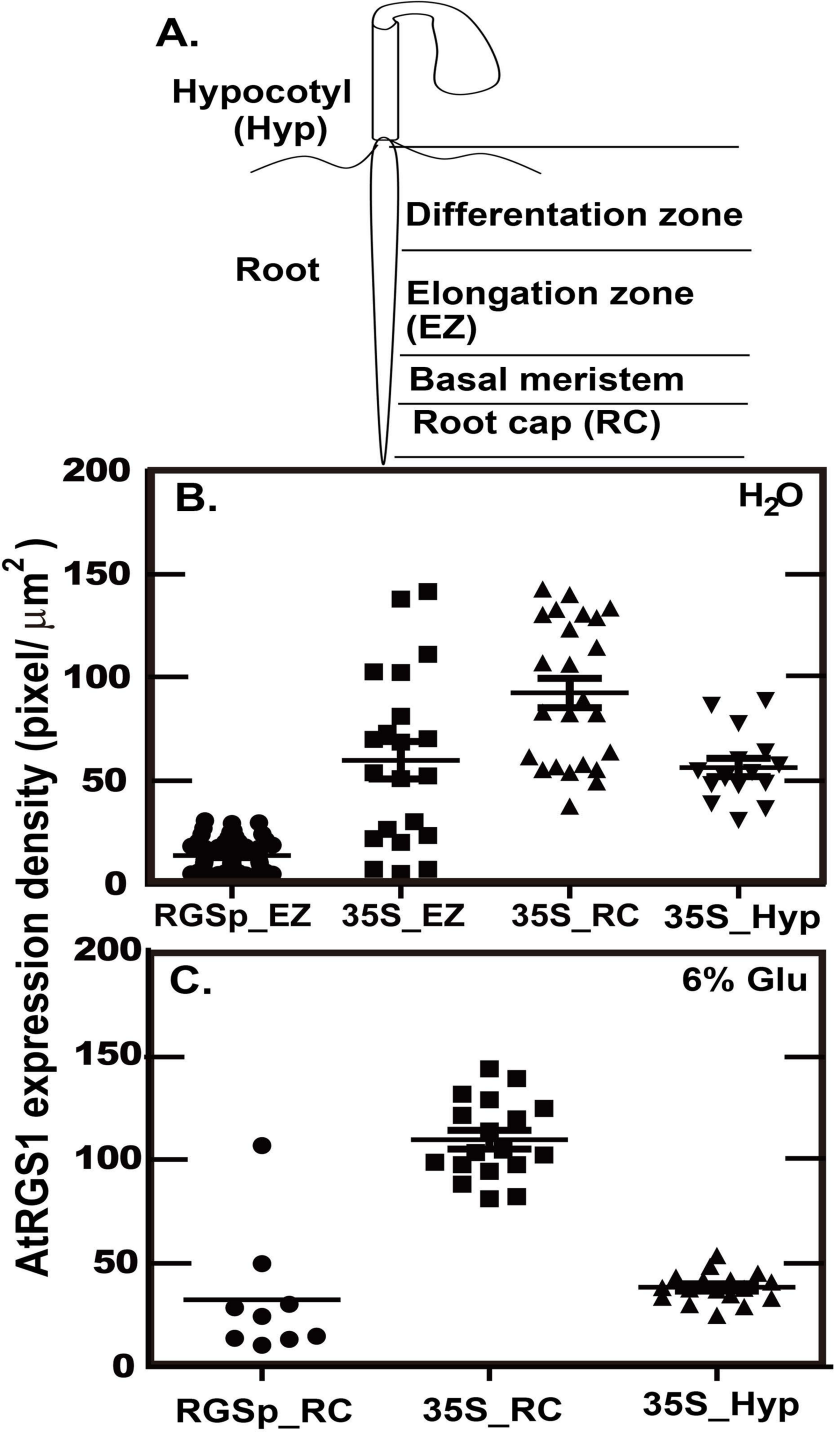
AtRGS1 level driven by the native and a strong viral gene promoter. AtRGS1 expression was driven by its native promoter (AtRGS1-YFP) or the 35S Califlower Mosaic Viral promoter (35S-YFP) in roots/hypocotyl cells treated with water for 30 minutes **(B)** and treated with 6% D-glucose for 1 hour **(C)**. Both of *AtRGS1-YFP* and *35S-YFP* have 1 gene copy. **(A).** The corresponding regions of the seedling are: hypocotyl (Hyp), elongation zone (EZ), and root cap (RC) in *Arabidopsis*. **(B).** Under water treatment, the AtRGS1 expression density of 35S-YFP in the elongation zone (35S_EZ), root cap (35S_RC), and hypocotyl (35S_Hyp) is around 4 to 6 fold of the AtRGS1 level of AtRGS1-YFP in elongation zone (RGSp_EZ). **(C).** Under 6% D-glucose treatment for 1 hour, both AtRGS1 expression density of AtRGS1-YFP and 35S-YFP in the root cap increases, but the ratio between RGSp_RC and 35S_RC is still around 4 fold. Note that the 6% D-glucose treatment does not change the AtRGS1 expression density of 35S-YFP in hypocotyl.

In order to distinguish the relative expression level, the following method and standard was adopted: the AtRGS1 level was measured to determine whether over expressing AtGRS1 altered the AtRGS1 level. In Fig 6, we measured the AtRGS1 level in the root using the native AtRGS1 promoter (RGSp, Fig 6) and compared this to expression at different locations of the root with the 35S promoter (35S, Fig 6). With two exceptions, the native AtRGS1 gene promoter (RGSp, Fig 6) was insufficient to detectably drive expression of AtRGS1-YFP. However, YFP fluorescence in some cells in the elongation zone and the root cap (Fig 6A) were detectable. This enabled estimation of the relative level of endogenous AtRGS1 to the amount of AtRGS1-YFP driven by the 35S promoter in the stable lines (35S, Fig 6B and 6C) under water and 6% glucose treatment, respectively. The ratio of AtRGS1 level between 35S-YFP at any region (i.e., root cap, elongated zone, and hypocotyl) and AtRGS1-YFP at the elongated zone and root cap was ~4. The later assay utilized the native level of AtRGS1 while the former utilized approximately a four-fold higher level of AtRGS1 (in the form of the AtRGS1-YFP reporter) shown in Fig 6B and 6C.

## Discussion

We tested the hypothesis that the AtRGS1 pool size modulates D-glucose-induced AtRGS1-YFP endocytosis, a cellular marker for sustained G protein activation (Fig 1). Our model predicted a nonlinear relationship between the AtRGS1 level and AtRGS1-YFP endocytosis (Fig 2); specifically that the system was less sensitive to glucose treatment when the system had higher AtRGS1 levels.

Changes in AtRGS1 level may occur in nature because we showed experimentally that the AtRGS1 level changes with NaCl and D-glucose treatments (Figs 4 and 5). This is a newly-discovered mechanism by which plant cells control glucose responsiveness through the G protein pathway. Our findings also provide a mechanism by which the nutrient state of the cell affects important signaling pathways. For example, glucose via the G protein signaling pathway improves plant survival under salt stress [21]. Glucose also enhances hypocotyl and root elongation through the brassinosteroid pathway [23, 24], promotes defense against pathogens by the innate immunity pathway [25, 26], and affects root partitioning through the auxin pathway [27].

How do these changes affect the rapid dynamics of Dose-Duration Reciprocity? Because the change in AtRGS1 is small but significant over two hours (Figs 4 and 5), the early dynamics of the Dose-Duration Reciprocity model is unaffected and therefore, a closed-system model remains suitable for predicting the rapid changes in the model components. However, over longer time spans such as the course of a solar day, the changes in the amount of AtRGS1 are expected to be in the range that we show here to affect the sensitivity. Over a long period, Does-Duration Reciprocity is better described mathematically by an open-system model.

## Supporting information

S1 Fig Comparison of the proportion of internalized AtRGS1 in wild type, *wnk*8/10, and *wnk*1 null mutants. **(A)**, 2% glucose. Wildtype, black solid (*C*1 = 4×10^4^ molecules) and dotted (*C*1 = 5×10^4^ molecules); *wnk8/10* mutant, blue solid (*C*1 = 4×10^4^) and dotted (*C*1 = 5×10^4^) curves represent the proportion of internalized AtRGS1 after 2% D-glucose treatment over 40 minutes. (**B**) 6% glucose. Wildtype, black solid (*C*1 = 4×10^4^ molecules) and dotted (*C*1 = 5×10^4^ molecules); *wnk1* mutant, magenta solid (*C*1 = 4×10^4^) and dotted (*C*1 = 5×10^4^) curves represent the proportion of internalized AtRGS1 after 6% D-glucose treatment over 120 minutes.
(TIF)

S2 Fig Heat map of proportion of internalized AtRGS1 in *wnk* mutants under different AtRGS1 levels. **This figure supports Fig 3.** This figure shows the heat map of the proportion of internalized AtRGS1 in the *wnk*8/10 null mutant **(A-C)** and *wnk*1 null mutant **(G-I)**, under water (top row), 2% (middle row)**,** and 6% (bottom row) D-glucose treatment, when the AtRGS1 level *C*1 is between [3.7×10^4^, 6.8×10^4^] molecules. The color bar shown at the top represents the proportion of internalized AtRGS1 value ranging from 0.00 (blue) to 1.00 (yellow). Similar to wild type in Fig 2, the proportion of internalized AtRGS1 is nonlinearly dependent on the AtRGS1 level. **(D-F)**. The time course is 60 minutes for the *wnk*8/10 null mutants with low, moderate, and high AtRGS1 level in (A-C). **(J-L)**. The time series within 60 minutes of the *wnk*1 null mutants with low, moderate, and high AtRGS1 level in (G-I). In (D-F) (resp. (J-L)), the solid curves, dotted curves, and dashed curves represent the solid line (i.e., C1 = 4×10^4^ molecules), dotted line (i.e., C1 = 5×10^4^ molecules), and dashed lines (i.e., C1 = 6×10^4^ molecules) in (A-C) (resp. (G-I)).
(TIF)

S3 Fig Sustained AtRGS1 level (*C*1 in the model) under flg22 condition. This figure shows the change in AtRGS1 intensity after treatment with 100 mM flg22 treatment. Because the effect from flg22 occurs quickly (around 10 minutes), the image was taken at 1 minute and 30 minutes at the same location of the same seedlings. After 100 mM flg22 treatment, the mean of AtRGS1-YFP intensity increased from 25 pixel/μ*m*^2^ to 29.1 pixel/μ*m*^2^, but the AtRGS1-YFP intensity is not significantly different over 30 minutes (p-value is around 0.02). For these experiments, AtRGS1-YFP was driven by the 35S viral promoter.
(PDF)

S1 File MATLAB code for pixel intensity measurement. This MATLAB code is used for the average pixel intensity of the images taken from light sheet fluorescence microscopy. The function find_avg_intensity(fileName) is used to calculate the average intensity of the image specified in fileName after removing the image background with the default intensity threshold 0.1 for separating signal from background. The function find_avg_intensity(fileName,TH) calculate the same thing as find_avg_intensity(fileName), besides the intensity threshold can be adjusted by changing the value of TH. The return values of both functions are the average pixel intensity of the region of the image above the threshold value (default setting 0.1 for find_avg_intensity(fileName) and TH for find_avg_intensity(fileName,TH)).
(PDF)
