# Supplementary figures and images for: Dose-Duration Reciprocity for G protein activation: Modulation of kinase to substrate ratio alters cell signaling
Source: PLoS One. 2017 Dec 29;12(12):e0190000. doi: 10.1371/journal.pone.0190000 (PMC5747438; doi:10.1371/journal.pone.0190000)

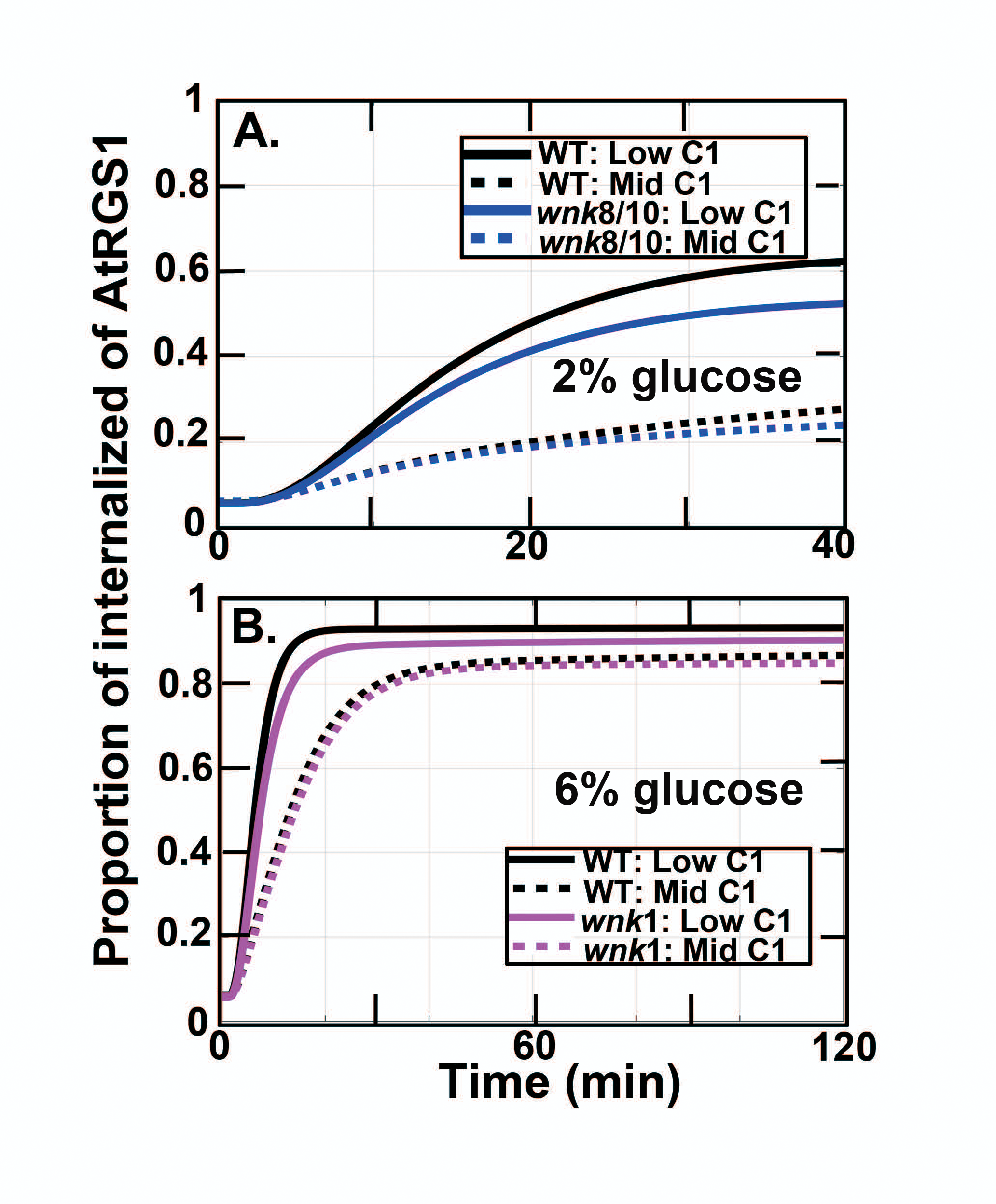

Supplement: S1 Fig — (A), 2% glucose. Wildtype, black solid (C1 = 4×10^4^ molecules) and dotted (C1 = 5×10^4^ molecules); wnk8/10 mutant, blue solid (C1 = 4×10^4^) and dotted (C1 = 5×10^4^) curves represent the proportion of internalized AtRGS1 after 2% D-glucose treatment over 40 minutes. (B) 6% glucose. Wildtype, black solid (C1 = 4×10^4^ molecules) and dotted (C1 = 5×10^4^ molecules); wnk1 mutant, magenta solid (C1 = 4×10^4^) and dotted (C1 = 5×10^4^) curves represent the proportion of internalized AtRGS1 after 6% D-glucose treatment over 120 minutes. (TIF) [file pone.0190000.s001.tif]

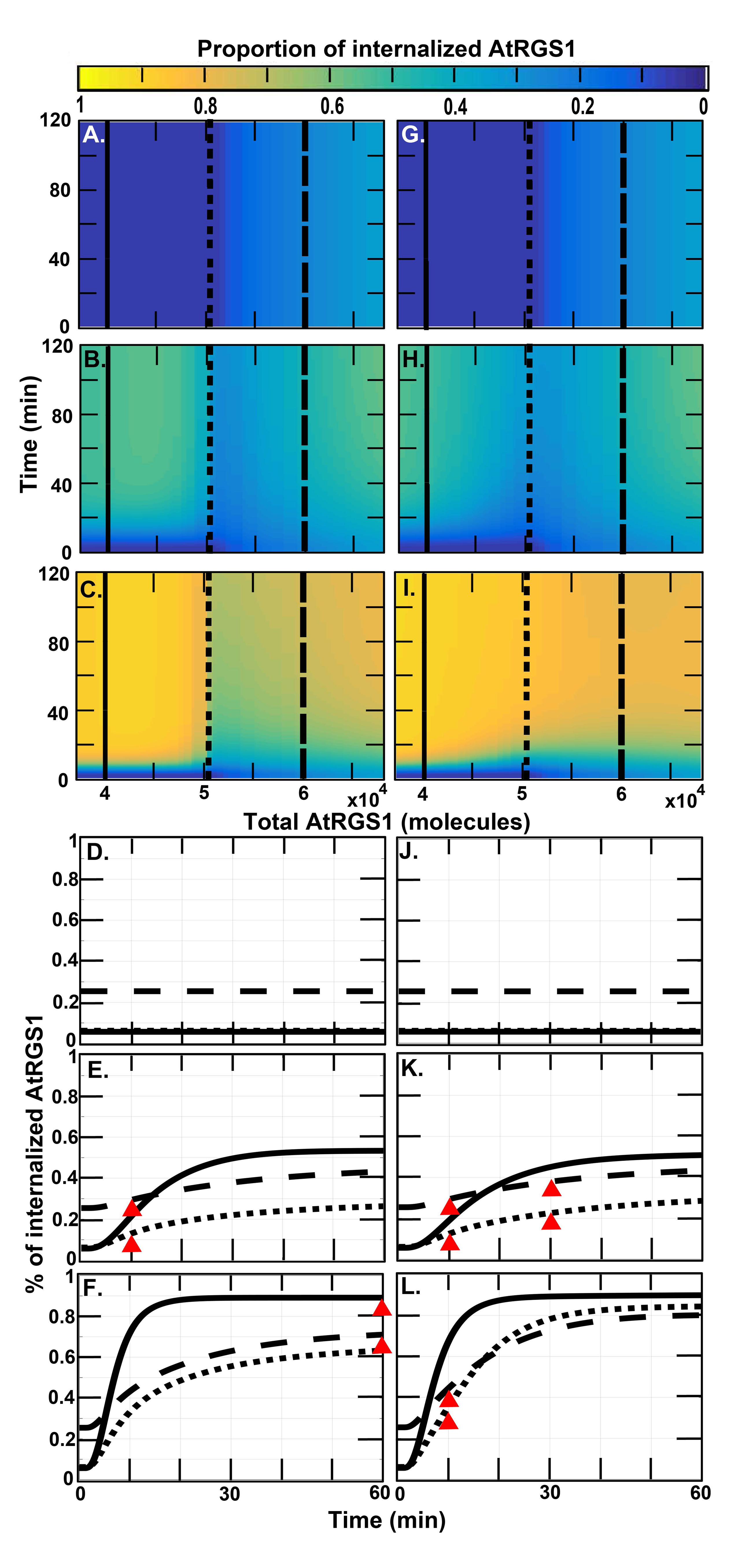

Supplement: S2 Fig — This figure supports Fig 3. This figure shows the heat map of the proportion of internalized AtRGS1 in the wnk8/10 null mutant (A-C) and wnk1 null mutant (G-I), under water (top row), 2% (middle row), and 6% (bottom row) D-glucose treatment, when the AtRGS1 level C1 is between [3.7×10^4^, 6.8×10^4^] molecules. The color bar shown at the top represents the proportion of internalized AtRGS1 value ranging from 0.00 (blue) to 1.00 (yellow). Similar to wild type in Fig 2, the proportion of internalized AtRGS1 is nonlinearly dependent on the AtRGS1 level. (D-F). The time course is 60 minutes for the wnk8/10 null mutants with low, moderate, and high AtRGS1 level in (A-C). (J-L). The time series within 60 minutes of the wnk1 null mutants with low, moderate, and high AtRGS1 level in (G-I). In (D-F) (resp. (J-L)), the solid curves, dotted curves, and dashed curves represent the solid line (i.e., C1 = 4×10^4^ molecules), dotted line (i.e., C1 = 5×10^4^ molecules), and dashed lines (i.e., C1 = 6×10^4^ molecules) in (A-C) (resp. (G-I)). (TIF) [file pone.0190000.s002.tif]

AtRGS1 fluorescence density

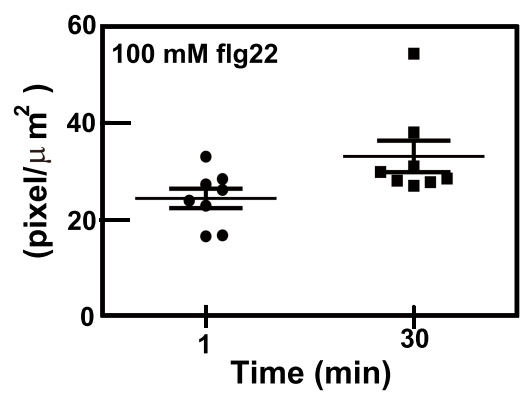

Supplement: S3 Fig — This figure shows the change in AtRGS1 intensity after treatment with 100 mM flg22 treatment. Because the effect from flg22 occurs quickly (around 10 minutes), the image was taken at 1 minute and 30 minutes at the same location of the same seedlings. After 100 mM flg22 treatment, the mean of AtRGS1-YFP intensity increased from 25 pixel/μm2 to 29.1 pixel/μm2, but the AtRGS1-YFP intensity is not significantly different over 30 minutes (p-value is around 0.02). For these experiments, AtRGS1-YFP was driven by the 35S viral promoter. (PDF) [file pone.0190000.s003.pdf]
