# Supplementary material for: Dose-Duration Reciprocity for G protein activation: Modulation of kinase to substrate ratio alters cell signaling
Source: PLoS One. 2017 Dec 29;12(12):e0190000. doi: 10.1371/journal.pone.0190000 (PMC5747438; doi:10.1371/journal.pone.0190000)
Supplement: S1 File — This MATLAB code is used for the average pixel intensity of the images taken from light sheet fluorescence microscopy. The function find_avg_intensity(fileName) is used to calculate the average intensity of the image specified in fileName after removing the image background with the default intensity threshold 0.1 for separating signal from background. The function find_avg_intensity(fileName,TH) calculate the same thing as find_avg_intensity(fileName), besides the intensity threshold can be adjusted by changing the value of TH. The return values of both functions are the average pixel intensity of the region of the image above the threshold value (default setting 0.1 for find_avg_intensity(fileName) and TH for find_avg_intensity(fileName,TH)). (PDF) [file pone.0190000.s004.pdf]

Matlab Code for pixel intensity measurement

```
function avg_intensity = find_avg_intensity(fileName,TH)

% find_avg_intensity(fileName) calculates the average intensity of the
% image specified in fileName after removing the image background. Default
% intensity threshold for separating signal from background is 0.1.

% find_avg_intensity(fileName,TH) calculates the average intensity of the
% image specified in fileName after removing the image background. The
% intensity threshold for separating signal from background is the one
% speficied in TH.

% find_avg_intensity returns the average pixel intensity of the region of
% the image above the threshold value.

% Example 1:
% avg_intensity = find_avg_intensity('fileName.tif',0.05)

% Example 2:
% avg_intensity = find_avg_intensity('fileName.tif')


if nargin < 1
    error('Not enough input arguments')
elseif nargin == 1
    TH = 0.1;
end

I = im2double(rgb2gray(imread(char(fileName))));
Ilog = im2bw(I,TH);
Iroi = I.*Ilog;

s = size(I);

count = 0;
sum = 0;
for row=1:s(1)
```

```
        for col=1:s(2)
            if~(Iroi(row,col)==0)
                count = count+1;
                sum = sum+Iroi(row,col);
            end
        end
    end
    avg_intensity = sum/count;
    imshow(Iroi);
    disp(['The sum pixel count for regions of interest is ' num2str(sum)]);
    disp(['The average pixel count for regions of interest is ' num2str(avg_intensity)]);
    disp(['The pixel count for regions of interest is ' num2str(count)]);

    end
```
